# Statement of Retraction: The role of autophagy-related proteins in the pathogenesis of neuromyelitis optica spectrum disorders

**DOI:** 10.1080/21655979.2026.2667602

**Published:** 2026-05-21

We, the Editors and Publisher of the journal *Bioengineered*, have retracted the following article from publication.

Guo, H. L., Shen, X. R., Liang, X. T., & Li, L. Z. (2022). The role of autophagy-related proteins in the pathogenesis of neuromyelitis optica spectrum disorders. *Bioengineered*, 13(6), 14329–14338. https://doi.org/10.1080/21655979.2022.2084273

Since publication, significant concerns have been raised about the integrity of the data and reported results in the article. Specifically, concerns were raised about alleged image duplication with similar images from an unrelated article: 

• Figure 4a ß-actin band, appears to have been duplicated with a similar image in Figures 4A and 5A from Xu et al, 2015 (https://doi.org/10.3892/mmr.2014.2683)  

• Figure 4a beclin 1 band, appears to have been duplicated with a similar image in Figure 5A from Xu et al, 2015 (https://doi.org/10.3892/mmr.2014.2683)  

When approached for an explanation, the authors have not responded to our queries and so these serious concerns remain.  

As verifying the validity of published work is core to the integrity of the scholarly record, we are therefore retracting the article. The authors have been informed of this decision.

We have been informed in our decision-making by our policy on publishing ethics and integrity and COPE guidelines.  

The retracted article will remain online to maintain the scholarly record and will be digitally watermarked on each page as ‘Retracted’.  

